# Dermato-neurologisches Syndrom – eine akute und lebensbedrohliche Komplikation des Skleromyxödems Arndt-Gottron

**DOI:** 10.1007/s00105-023-05159-w

**Published:** 2023-06-07

**Authors:** Kai-Philipp Linse, Alexander Enk, Jochen Hoffmann

**Affiliations:** Abteilung Dermatologie, Venerologie und Allergologie, Universitätsklinikum Heidelberg, Im Neuenheimer Feld 440, 69120 Heidelberg, Deutschland

**Keywords:** Intravenöse Immunglobuline, SARS-CoV‑2, Influenza A, Neurologische Komplikation, Muzinablagerungen, Intravenous immunoglobulins, SARS-CoV‑2, Influenza A, Neurological complications, Mucinosis

## Abstract

Das Skleromyxödem Arndt-Gottron ist die systemische Variante des Lichen myxoedematosus. Kennzeichnend sind Muzinablagerungen im Bereich der Dermis mit Ausbildung von Papeln und flächigen Indurationen. Der Verlauf ist häufig chronisch progredient und extrakutane Manifestationen bzw. Komplikationen sind möglich. Meist liegt eine monoklonale Gammopathie vor. Die Pathogenese ist unbekannt. Als effektive Therapieoption gelten hochdosierte intravenöse Immunglobuline (IVIg). Wir berichten über eine Patientin mit Skleromyxödem, bei der im Rahmen einer SARS-CoV-2-bedingten Therapieunterbrechung ein infektgetriggertes dermato-neurologisches Syndrom auftrat. Eine ähnliche Episode ereignete sich bereits 2 Jahre zuvor im Rahmen einer Influenza-A-Infektion gegen Ende des IVIg-Infusionsintervalls. Bei dem dermato-neurologischen Syndrom handelt es sich um eine besonders schwerwiegende, potenziell lebensbedrohliche, akute neurologische Komplikation mit Fieberschüben, deliranter Symptomatik, epileptischen Anfälle und Koma.

## Abstract (en)

Scleromyxedema Arndt–Gottron is the systemic variant of lichen myxedematosus in which mucin accumulation occurs in the dermis. The disease is usually chronically progressive and extracutaneous manifestations or complications are possible. The pathogenesis is unknown and the disease is usually associated with monoclonal gammopathy. High-dose intravenous immunoglobulins (IVIg) are considered to be an effective therapy. We report the case of a patient who developed dermato-neuro syndrome following an interruption of IVIg treatment and a SARS-CoV‑2 infection. A similar episode occurred 2 years earlier in association with an influenza A infection. Dermato-neuro syndrome is a potentially lethal neurological complication which is characterized by fever, delirium, convulsions, and coma.

## Anamnese

Wie berichten über eine 70-jährige Patientin, die aufgrund eines langjährig bekannten Skleromyxödems Arnd-Gottron eine dauerhafte Behandlung mittels IVIg (2 g/kg Körpergewicht) im Intervall von zuletzt fünf Wochen erhielt. Begleitend waren ein seit vielen Jahren stabil verlaufendes „smoldering myeloma“, eine arterielle Hypertonie und ein Diabetes mellitus Typ II bekannt. Aufgrund einer SARS-CoV-2-Infektion im Familienkreis konnte eine anstehende IVIg-Behandlung nicht im planmäßigen Intervall erfolgen. Im weiteren Verlauf infizierte sich die Patientin und entwickelte einen generalisierten Krampfanfall mit konsekutiver Todd’scher Parese und deliranter Symptomatik. Die Patientin hatte zwei Impfungen gegen SARS-CoV‑2 erhalten, wobei die letzte Impfung sieben Monate vor der Infektion erfolgte. Eine ähnliche Episode ereignete sich bereits zwei Jahre zuvor im Rahmen einer Influenza-A-Infektion gegen Ende des Infusionsintervalls von damals sechs Wochen.

## Befund

Aufgrund ausgeprägter deliranter Symptomatik mit Verwirrtheit, optischen Halluzinationen und Dysarthrie erfolgte eine notfallmäßige stationäre Aufnahme in der Neurologie. Eine zerebrale Blutung oder Ischämie konnte computertomographisch ausgeschlossen werden.

## Diagnose

In Zusammenschau von Klinik und Anamnese wurde nach Ausschluss infektiöser und metabolischer Ursachen die Diagnose eines dermato-neurologischen Syndroms im Rahmen eines Skleromyxödems bei SARS-CoV-2-Infektion und gleichzeitig nicht erhaltener IVIg-Gabe gestellt.

## Therapie und Verlauf

Die Patientin wurde intensivmedizinisch betreut und musste bei zunehmender Vigilanzminderung schutzintubiert sowie zeitweise maschinell beatmet werden. Es erfolgte eine IVIg-Behandlung (2 g/kg Körpergewicht) und eine antiepileptische Therapie mit Levetiracetam wurde initiiert. Die Patientin sprach erfreulicherweise gut auf die IVIg-Therapie an. Der neurologische und allgemeine Zustand der Patientin stabilisierten sich zusehends, sodass die Patientin im weiteren Verlauf entlassen und einer ambulanten Rehamaßnahme zugeführt werden konnte.

## Diskussion

**Figure 1:**
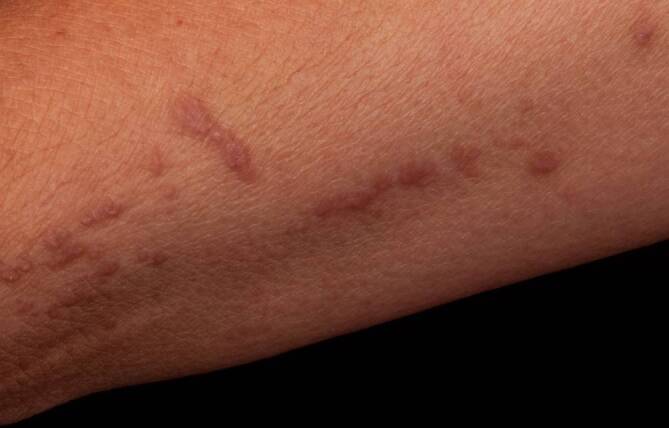

Das Skleromyxödem ist die systemische Variante des Lichen myxoedematosus. Kennzeichnend für die Erkrankung sind Muzinablagerungen im Bereich der Dermis mit Papeln und flächiger Induration der Haut (Abb. 1), zunehmender Einschränkung der Beweglichkeit und unvorhersehbarem, häufig chronisch progredientem Verlauf. Zudem sind zahlreiche extrakutane Manifestationen bzw. Komplikationen beschrieben (v. a. kardiovaskulär, hämatologisch und neurologisch), wobei die Hautveränderungen den übrigen Manifestationen in der Regel vorausgehen. Die Pathogenese ist unbekannt. Die Erkrankung geht in der Regel mit einer monoklonalen Gammopathie einher, wobei der kausale Zusammenhang zwischen der Paraproteinämie und den Hauterscheinungen unklar ist [1, 2]. Eine besonders schwerwiegende, potenziell lebensbedrohliche, akute neurologische Komplikation stellt das dermato-neurologische Syndrom dar, das durch Fieberschübe, delirante Symptomatik, epileptische Anfälle und Koma gekennzeichnet ist [1, 3, 4]. Die Pathogenese des dermato-neurologischen Syndroms ist ebenfalls ungeklärt. Es werden eine erhöhte Blutviskosität oder eine Leukozytenaggregation infolge der Paraproteinämie diskutiert [4, 5]. Möglicherweise spielt auch eine, Interleukin(IL)-6-bedingte, Ig(Immunglobulin)G-vermittelte Entzündungsreaktion bei gestörter Blut-Hirn-Schranke eine Rolle [6]. Als First-line-Therapie des Skleromyxödems und seiner neurologischen Komplikationen haben sich IVIg etabliert [1, 7].

Wir berichten über ein zweimalig aufgetretenes, infektgetriggertes dermato-neurologisches Syndrom vor dem Hintergrund einer verpassten IVIg-Infusion bzw. gegen Ende des Infusionsintervalls. Ein möglicher Zusammenhang zwischen ausgesetzter IVIg-Therapie bzw. verlängerten Therapieintervallen und dem Auftreten neurologischer Komplikationen wurde bereits zuvor beschrieben [3, 7]. Ebenso existieren Fallberichte, in denen ein klarer zeitlicher Zusammenhang zwischen akuten respiratorischen Virusinfektionen und dem Auftreten eines dermato-neurologischen Syndroms beschrieben wird. So existieren bislang vier Fälle, in denen eine Influenza-A-Infektion, und ein Fall, in dem eine SARS-CoV-2-Infektion in Verbindung mit einem dermato-neurologischen Syndrom nachgewiesen wurde. Andere Ursachen für die neurologische Symptomatik, insbesondere eine virusbedingte Enzephalitis, konnten in diesen Fällen nicht nachgewiesen werden. Es wurde deshalb jeweils die Diagnose eines möglicherweise infektgetriggerten dermato-neurologischen Syndroms gestellt [8–10]. Es ist bekannt, dass IL‑6 als proinflammatorisches Zytokin bei viralen Infektionen wie Influenza A oder SARS-CoV‑2 vermehrt synthetisiert und freigesetzt wird [8]. Zudem konnte gezeigt werden, dass sowohl der Liquor- als auch der Serum-IL-6-Spiegel bei Skleromyxödem-assoziierter Enzephalopathie deutlich erhöht sind [1, 6, 8]. Diese Beobachtungen deuten auf einen möglichen Zusammenhang akuter respiratorischer Virusinfektionen, insbesondere SARS-CoV‑2 und Influenza A, mit der Entstehung eines dermato-neurologischen Syndroms beim Skleromyxödem hin [8, 9].

## Fazit für die Praxis

- Im vorliegenden Fall ist der zeitliche Verlauf suggestiv dafür, dass die Kollision des respiratorischen Infekts mit der Therapieunterbrechung bzw. dem Ende des Infusionsintervalls jeweils ursächlich für das Auftreten des dermato-neurologischen Syndroms war.
- Die IVIg-Behandlung sollte nach Möglichkeit im planmäßigen Intervall erfolgen.
- Bei Auftreten eines dermato-neurologischen Syndroms sollte neben dem Ausschluss neurotroper Infektionen stets auch auf andere Erreger, insbesondere respiratorische Virusinfektionen, getestet werden.
